# Food Matrices Affect the Peptides Produced during the Digestion of *Arthrospira platensis*-Based Functional Aliments

**DOI:** 10.3390/nu13113919

**Published:** 2021-11-01

**Authors:** Giuliana Donadio, Valentina Santoro, Fabrizio Dal Piaz, Nunziatina De Tommasi

**Affiliations:** 1 Department of Pharmacy, University of Salerno, Via Giovanni Paolo II, 84084 Fisciano, Italy; gdonadio@unisa.it (G.D.); vsantoro@unisa.it (V.S.); detommasi@unisa.it (N.D.T.); 2 Department of Clinic Pharmacology, University Hospital San Giovanni di Dio e Ruggi d’Aragona, Via San Leonardo, 84125 Salerno, Italy; 3 Department of Medicine, Surgery and Dentistry, University of Salerno, Via S. Allende, 84082 Baronissi, Italy

**Keywords:** Spirulina, simulated gastro-intestinal digestion, mass spectrometry, bioactive peptides, phycocyanin

## Abstract

*Arthrospira platensis* (Spirulina) has been credited with multiple beneficial effects, many of which are attributed to bioactive peptides produced during the gastrointestinal digestion of this micro-alga. Many Spirulina-based nutraceuticals have been produced, and numerous functional foods enriched with Spirulina are available on the market. These are subjected to checks aimed at verifying the amount of algae actually present, but few studies relating to the bioavailability of the bioactive compounds in these products have been carried out. However, such investigations could be very important to elucidate the possible critical effects exerted by food matrices on protein digestion and bioactive peptide production. Here, in order to assess the suitability of Spirulina-enriched foods as a source of potentially bioactive peptides, a simulated digestion protocol was used in combination with mass spectrometry quantitative analysis to analyze functionalized pasta and sorbets. In the case of the pasta enriched with Spirulina, the production of peptides was quite similar to that of the Spirulina powder. On the other hand, the type of fruit present in the food matrix influenced the digestion of Spirulina inside the sorbets. In particular, the high concentration of protease inhibitors in kiwifruit drastically reduced the production of peptides from Spirulina in kiwi sorbet.

## 1. Introduction

*Arthrospira platensis*, better known as “Spirulina platensis” or merely Spirulina, is a cyanobacterium used for centuries as a food by different populations and only rediscovered in recent years. Many different health benefits have been attributed to cyanobacteria, due to their high content of amino acids [1], proteins [2], vitamins [3,4,5], minerals, essential fatty acids [6,7], and β-carotene [8,9]. Based on this, Spirulina can be considered a sort of superfood; in fact, in 1992 the World Health Organization (WHO) defined this microalga as the “best food for the future” and NASA [10] believes it is an excellent compact food for space travel, because a small amount can provide a wide range of nutrients [2]. Several studies have demonstrated the primary role played by the proteins and peptides of Spirulina in its beneficial effects on human health. Specifically, this microalga is an excellent source of phycobiliproteins [11,12]. The amino acid composition of these proteins suggests the existence of significant peptides encrypted within their sequences, which can exert different bioactivities after being released by the digestive processes [13,14,15]. The effects of bioactive peptides depend on their specific amino acid composition and sequence; these molecules have been credited with potential beneficial effects on the cardiovascular system (antihypertensive, antioxidant, antithrombotic, and hypocholesterolemic), nervous system (opioid), gastrointestinal system (mineral binding, anti-appetizing, and antimicrobial), and immune system (antimicrobial, immunomodulatory, and cytomodulatory) [16,17,18,19,20,21]. A number of bioactive peptides obtained from different kinds of enzymatic digestion of Spirulina have been described, most of which consist of small fragments of phycocyanins, typical of cyanobacteria. These proteins are water-soluble blue pigments found only in some species of blue-green microalgae, the concentration of which depends on the prevailing light conditions. The blue pigment has been related to several biological effects, potentially beneficial to human health; for example, orally administrated phycocyanins resulted in a significant decrease in the mortality of mice in which human liver tumor cells were implanted [22]. Furthermore, phycocyanins exert antioxidant and anti-viral effects, and should stimulate the immune system [4,23,24,25].

In light of the properties of this microalga, numerous nutraceutical products based on Spirulina have been proposed. Spirulina powder offers great potential use in the dairy industry to enrich products and to replace synthetic additives, such as dyes, stabilizers and emulsifiers. This microalga can also be added as a powder to increase the nutritional value of bread, resulting in products that show the color and the flavor of algae and contain a large amount of vitamins, microelements and phycocyanins. The addition of microalgae also improved water retention in the bread, thus increasing the long-term storage time of the product [26]. Therefore, an ever-increasing number of functional foods are available on the market: pasta, ice creams, health drink, sour milk and green tea, as well as various supplements in which Spirulina is used alone or in combination with other natural substances [27].

Currently, many of these products are subjected to quality controls [28,29] to verify the quantity of Spirulina, and to measure the concentration of carotenoids, total phenolic content or antiradical activity [4,5], but very few studies have been conducted to evaluate the effect of inclusion in different foods on the bioavailability of the bioactive molecules of the alga. Multiple factors can influence the bioavailability of active compounds, and in particular, of the peptides. The production of these molecules is mainly dependent on the digestive processes in the stomach and intestines, and can be, for example, significantly affected by the presence of other protein-rich foods; moreover, the incorporation of Spirulina in complex matrices can reduce its accessibility to enzymes. The aim of this research was therefore to evaluate the level of peptide production in the gastrointestinal digestion process of Spirulina-functionalized foods, and compare it with that observed for Spirulina powder in order to verify the efficacy of these products as a source of bioactive peptides. Therefore, a protocol was developed and optimized to simulate the enzymatic degradation that occurs in the gastrointestinal tract and the simulated digestion products were quantified through a mass spectrometry-based analytical method.

## 2. Materials and Methods

### 2.1. Materials

Spirulina powders, all sold as medium heat (40–50 °C) dried powders and certified 100% pure, were purchased from various companies, ITALGA Spirulina Italian produced by S.T.A.R. srl (Castel Baronia, Italy) (powder A), Neoalgae Micro Seaweeds Products (Gijon, Spain) (B) and MySuperfoods (Canterbury, UK) (C). Pasta enriched with Spirulina (NATOO Pasta BIO) had a concentration of algae of 3%. The sorbets, prepared by the “Gelati Alhoa” company, were composed of about 50% fruit pulp (kiwifruit, lemon, plum or apricot) and water, sugar, glucose-fructose syrup, dextrose and maltodextrins.

### 2.2. Gastrointestinal Simulated Digestion

**Figure 1:**
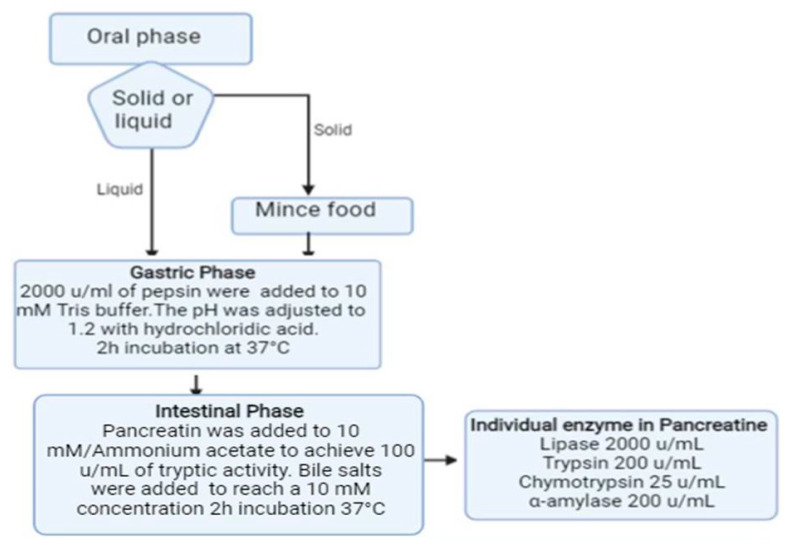
Schematic representation of the simulated digestion procedure set-up to study the production of peptides from Spirulina-containing aliments.

Simulated gastric fluid (SGF) and simulated intestinal fluid (SIF) were prepared according to the United States Pharmacopeia and Fu et al. [30]. Briefly, SGF was prepared by dissolving pepsin (Merk, Darmstadt, Germany) in 10 mM Tris buffer (tris-hydroxymethyl-aminomethane), 150 mM NaCl (pH 6.5) to a final concentration of 2000 u/mL. The pH was then adjusted to 1.2 with hydrochloric acid under stirring. To set up SIF, pancreatine (a mixture of lipase, trypsin, chymotrypsin and α- amylase) (Merk) was dissolved in 10 mM ammonium acetate to achieve 100 u/mL tryptic activity; bile salts (50% sodium cholate-50% sodium deoxycholate) (Merk) were then added to the mixture to reach a final concentration of 10 mM. Dissolution of the bile required thorough mixing at 37 °C for 30 min (Figure 1).

The first step of the digestion protocol (Figure 1) consisted in the incubation of an appropriate amount (50 mg of ground Spirulina powder and 1.5 g of pasta or sorbet) of the selected solid sample in 5 mL of SGF, at 37 °C for 120 min under stirring. The reaction was stopped by heating the mixture at 95 °C for 10 min and increasing the pH to 7.0. The solution was cooled at room temperature for 15 min and then mixed with 5 mL of SIF; the pH was adjusted to 7.0 with 0.1 M NaOH. Simulated intestinal digestion proceeded at 37 °C for 120 min, after which the mixture was heated at 95 °C for 10 min and the pH was lowered to 2. After centrifugation, the supernatant was collected and stored at −20 °C until use. Before LC-MS/MS analyses, the samples were filtered through a 0.22 µm filter. Spirulina A, B and C, as well as the selected functional foods, were subjected to the described protocol. All samples were analyzed at least in triplicate.

### 2.3. Qualitative Analysis

An LC-HRMS/MS method was setup to analyze the peptide profile of Spirulina samples after simulated gastrointestinal digestion.

The instrument configuration adopted was an Ultimate 3000 (Thermo Fisher Scientific, Waltham, MA, USA) UPLC system interfaced, via an ESI source, to a high-resolution mass analyzer (Q-exactive Orbitrap, Thermo Fisher Scientific). The peptides separation was performed on a C18 column (Luna C18, Phenomenex, 150 × 2.0 mm, 3 µm), using 0.1% (*v*/*v*) ultrapure water–formic acid as eluent A, 0.1% (*v*/*v*) ultrapure acetonitrile–formic acid/0.1% (*v*/*v*) ultrapure water–formic acid (50:50 *v*/*v*) as eluent B, and a gradient from 5% to 35% of B within 30 min, and from 35% to 70% of B within 10 min. The flow rate was 0.200 mL/min and the injection volume was 10 µL. MS data were acquired in positive ion mode. The method involved full-mass and data-dependent scan experiments. The capillary temperature was set at 320 °C, and the sheath gas and auxiliary gas flow rates were set at 35 and 15 arbitrary units, respectively. The source voltage was 3.0 kV and the mass resolution was set at 35,000. Mass spectra were acquired over an *m*/*z* range between 375 and 1500. To obtain sequence confirmation, MS and MS/MS data were analyzed by Mascot software (v2.5, Matrix Science, Boston, MA, USA) over the non-redundant Data Bank UniprotKB/Swiss-Prot (Release 2020_03). The parameter sets were: trypsin cleavage; methionine oxidation as a variable modification; a maximum of two missed cleavages; and false discovery rate (FDR), calculated by searching the decoy database, 0.05. 

### 2.4. Quantitative Analysis 

After the selection of the peptide markers, an MRM method was developed on an API6500 Q-Trap (ABSciex, Foster City, CA, USA) coupled with a NexeraX2 UHPLC apparatus (Shimadzu, Kyoto, Japan). A positive multiple-reaction monitoring (MRM) system was set up according to mascot and LC-HRMS/MS data (see Table S1 for the experimental details). A Luna Omega C18 column (Phenomenex, 100 × 2.1 mm, 1.6 μm) and a linear gradient from 5% to 30% of acetonitrile containing 0.1% formic acid (eluent B) in ten minutes were adopted to achieve peptides separation. The flow rate was 0.3 mL/min, and the injection volume was 10 μL. Angiotensin was used as an internal standard (monitored transition 516–263).

Quantitative data were measured as the ratio of the peak area of each peptide to that of angiotensin. All experiments were performed in triplicate and the data were reported as the mean of the three measurements ± standard deviation.

### 2.5. Method Performance 

To assess the suitability of the method to provide accurate results, identical samples (all consisting of 50 mg of Spirulina powder A, ground in a mortar for 10 min) were subjected to the entire simulated digestion and quantitative analysis of the peptides produced, three times on the same day (intraday precision) and then six more times in the next two days (interday precision). In addition, samples containing three different quantities of Spirulina A (20, 40 and 80 mg) were analyzed in triplicate to assess the linearity of the method.

### 2.6. Electrophoresis and Spectroscopic Analysis of Spirulina Protein Extracts 

Fifty milligrams of the three different Spirulina biomasses (A, B and C) were ground and incubated at room temperature with 5 mL of phosphate buffered saline (PBS) composed of 37 mM NaCl, 2.7 mM KCl, 8 mM Na_2_HPO_4_, and 2 mM KH_2_PO, pH 7.4) solution. After 1 h, the surnatants were collected and subjected to electrophoresis (SDS-PAGE) analysis, using a 12.5% polyacrylamide gel and pre-stained protein molecule weight markers (26 kDa–180 kDa) (Merk). The same extracts were centrifuged to eliminate any solid residue and their absorbance at 620 nm was measured to estimate the concentration of phycocianins [31].

### 2.7. Statistics

The quantitative data were subjected to statistical analyses aimed at defining the significant differences in peptides levels. Specifically, the data concerning the pasta samples (PS and P+S) were analyzed by the Student-*t* test (*n* = 3) and those of Spirulina powders A, B and C and sorbets were analyzed by the ANOVA test (*n* = 6). The observed differences were considered significant when *p* was ≤0.05 (*) or ≤0.01 (**)

## 3. Results and Discussion

The beneficial effects provided by Spirulina proteins depend on the production of bioactive peptides in the gastrointestinal digestion process of this alga. To compare the production of peptides from Spirulina powder and functional foods containing this microalga, we developed a bioanalytical protocol based on the use of a simulated two-step gastrointestinal digestion followed by quantitative mass spectrometry (MS) analyses of the digestion products (Figure 1). 

The digestion protocol was set up and optimized on Spirulina powder (powder A) and then used for all analyzed samples. The final protocol was as follows: 50 mg of powdered microalga were incubated at pH 2 in the presence of gastric proteases, and then further digested with pancreatic enzymes. The resulting mixture was analyzed by high-resolution MS in positive ion mode, without any purification treatment. The data obtained were processed using a specific bioinformatics tool. This procedure led to the identification of 61 peptides (Supplementary Materials, Table S1) belonging to both allo- and phycocianin proteins. Although there are numerous studies on Spirulina peptides, and also data on serine protease- or pepsin-catalyzed digestion of the proteins of this alga [32,33], a complete list of peptides produced in simulated gastrointestinal digestion of Spirulina was not available and many of the peptides we identified had never been described. It is therefore evident that this information will be very useful for future studies on Spirulina peptides.

In order to fine-tune a semi-quantitative analytical method that is rapid and easy to apply, it was necessary to select a limited number of these peptides, which was also representative of all protein species. Therefore, 18 peptides (Table 1) were chosen among the identified ones, taking care to have at least 4 peptides for each protein; further selection criteria were the abundance and specificity of the corresponding ions. Peptides including at least 5 amino acids were selected and their unique belonging to a single protein was evaluated by FASTA software. When possible, peptides for which biological activity has been reported were preferred: in fact, peptides 6, 9, 12, 15, 17 and 18 were suggested as putative dipeptidyl peptidase-IV inhibitors, possibly related to Spirulina anti-diabetic effects [34], while an anti-hypertensive action was shown for the tripeptide FEL included in peptides 13 and 16 [14]. 

**Figure 2:**
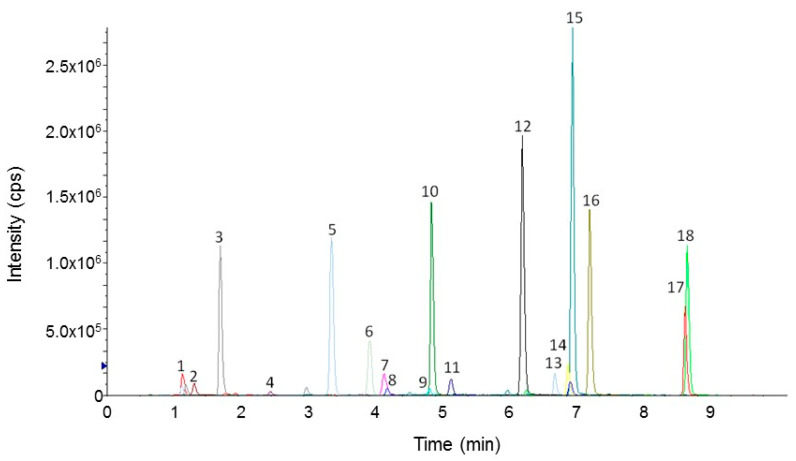
XIC of the peptide mixture obtained by the simulated digestion of 50 mg of Spirulina powder A.

After the selection of the peptide markers (Table 1), a specific and accurate MRM mass spectrometry method was developed, optimizing the chromatographic and mass spectrometry parameters. Figure 2 shows the extracted ion chromatogram (XIC) obtained from the LC-MS/MS analysis in MRM mode of the digestion products of Spirulina powder A. All observed peaks were well defined and easily integrated, thus allowing the correct measurement of their area.

The precision of the method was evaluated by analyzing three identical samples (50 mg of Spirulina powder A) in triplicate on the same day and over the following three days. In all cases, the area measured for all peptides was within the +/− 10% range. To confirm the suitability of the developed method to provide a dose-dependent response, 20, 40 and 80 mg of Spirulina powder A were analyzed. A linear trend was observed for 16 out of the 18 analyzed peptides. However, in the case of peptides 1 and 8, no increase in the corresponding signal area occurred as the concentration increased, probably due to characteristic kinetics of the digestion processes. Therefore, these peptides were taken into account only as markers for qualitative evaluations.

**Figure 3:**
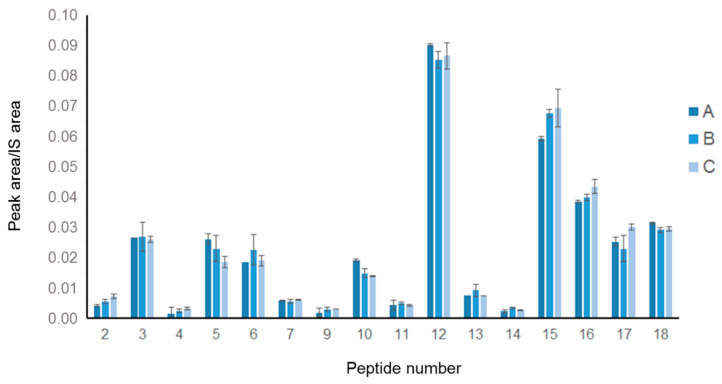
Quantitative analysis of the marker peptides in the LC-MS/MS analysis of three different Spirulina samples (A, B, C). Measurements were performed in triplicate on two different samples of each powder (*n* = 6): the graphs report the average areas and standard deviations. No significant differences between the abundance of peptides from the three samples were observed.

Moreover, to evaluate the reproducibility of our approach, three different commercially available 100% pure Spirulina powders (i.e., Spirulina A, B and C) were subjected to the entire procedure and the results obtained were compared. Preliminarily, the proteins extracted from 1 g of each powder were analyzed by SDS-PAGE followed by Coomassie blue staining (Supplementary Materials, Figure S1a), to evaluate any significant differences in the protein content of the three samples. The patterns of the detected bands and the results of the densitometric analysis of the gel were indicative of substantial similarity between the samples. Furthermore, the UV-Vis absorbance (λ range 400–750 nm) of the protein extracts was measured to compare the total quantities of phycocyanin in the three samples; again, the acquired spectra (Supplementary Figure S1b) showed that comparable amounts of protein were obtained from the three Spirulina powders. Based on these results, Spirulina A, B and C were subjected to simulated digestion followed by LC-MS/MS analysis, as described above. The data obtained for the three samples were very similar, from a qualitative and quantitative point of view (Figure 3). The 18 peptide markers were detected in all samples and their quantities were clearly comparable, as no statistically significant difference was measured. These results confirmed that the analytical protocol was sufficiently reproducible to be used as a suitable tool for studying peptide production from digesting Spirulina-containing samples.

Spirulina-enriched pasta was then investigated using the described protocol. Pasta is a food with an important role in the human diet and it is popular for its easy handling, storage and preparation; recently, different types of pasta with added microalgae and cyanobacteria are becoming more popular [35,36]. Published results have highlighted the positive effects of inclusion in food matrixes in improving the bioavailability of Spirulina carotenoids and polyphenols [37]. Moreover, Fradinho et al. [38] evaluated the potential of Spirulina for use in gluten-free pasta, reporting a high content of phenolic compounds, chlorophylls and carotenoids in this functional food, with no structural changes occurring in the pasta after incorporation of the seaweed. However, to the best of our knowledge, no information has been reported regarding the production of potentially bioactive peptides from these functional foods. 

To evaluate the impact of adding microalga to the pasta on the production of Spirulina peptides, a commercially available paste sample (1.5 g) with a declared Spirulina content of 3% (PS) was analyzed. It was cooked (10 min in water at 100 °C), freeze-dried and subjected to the simulated digestion process described above, followed by MRM-based LC-MS/MS analysis. The same protocol was also performed on a sample of microalga-free pasta (P0) and on one (P+S) to which, immediately before cooking, a quantity of Spirulina powder A equal to that theoretically present in PS was added. 

**Figure 4:**
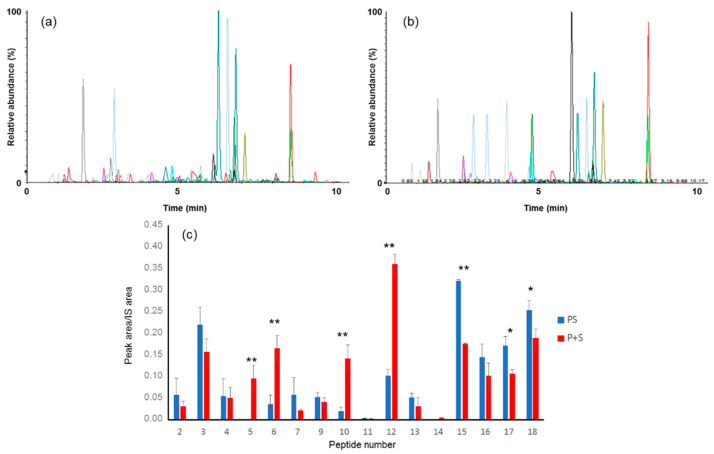
XIC obtained by the analysis of PS (**a**) and P+S (**b**) subjected to simulated gastro-intestinal digestion. The experiment was performed in triplicate (*n* = 3) and the average area measured for each peptide in the two samples were compared (**c**). * *p* ≤ 0.05 and ** *p* ≤ 0.01 indicate a significant difference between the peptide abundance in PS and P+S samples.

None of the peptide markers were detected in P0, thus confirming that the selected peptides were only observable in samples in which Spirulina was present. MRM chromatograms obtained by analyzing the samples from PS and P+S (Figure 4a,b) showed almost all of the peptides, confirming that these compounds were actually produced in the simulated digestion of both these foods. However, a comparison between the peak areas measured for the different markers in the two samples (Figure 4c) revealed that the incorporation of the microalga into the pasta matrix produced some perturbation in the abundances of the generated peptides. In fact, in the digestion of P+S a lower quantity of many peptides—in particular of the more hydrophobic ones (15–18)—was produced, compared to what was observed in the case of PS (Figure 4). This could depend on the prolonged boiling of the microalga powder alone, while Spirulina inside the dough was partially protected from thermal degradation. On the other hand, compounds 5, 6, 10 and 12 were much less abundant in the digested PS sample; intriguingly, these four peptides all come from the C-phycocyanin beta protein. The underlying causes of these results are not easily conceivable. Possibly, this protein, which is intrinsically prone to intermolecular interaction as it is involved in a multimeric complex with the alpha subunit of C-phycocyanin, binds efficiently to pasta proteins (albumins, globulins, gliadins and glutenins), thus being partly protected by proteolysis.

However, from a functional point of view, these results suggest that most of the potential beneficial effects of microalgae peptides should be retained in Spirulina-enriched pasta, regardless of the different production levels of the individual molecules. Indeed, the lower abundance of some species could be compensated for by the increase in that of others. For example, some potential anti-diabetic effects of Spirulina [34] have been related to peptides 6 and 12, whose amount was quite low following PS digestion, but also to compound 15, which is highly produced in the digestion of functionalized pasta.

The same approach used to study PS was then applied to the analyses of sorbets enriched with Spirulina. The sorbet is a food matrix clearly different from pasta, also from a technological point of view, Spirulina powder is added to sorbets at the end of their production process. Moreover, while in the case of pasta, Spirulina is subjected to cooking before being ingested, the seaweed in the sorbet only experiences storage at low temperatures. Sorbets were chosen instead of ice cream to reduce the complexity of the matrix, thus preventing possible interference. Ice cream is a frozen dessert made from a custard made of milk, cream, sweetener, and often egg yolks, while sorbet is a parfait composed only of fruit purée. Therefore, thanks to the high content of fresh fruit, sorbets have a high content of vitamins and other compounds considered beneficial; however, the fortification of sorbet with nutrients or other bioactive substances is in great demand and widely supported in the current market.

Four different sorbet flavors, kiwifruit, plum, apricot, lemon were analyzed to assess whether a specific fruit could have a more relevant effect on Spirulina peptide production. Indeed, the producers have selected these fruits in order to create gustatory and visual harmony between the meeting of sorbet and Spirulina, without taking into account the effective beneficial effect of the combination.

**Figure 5:**
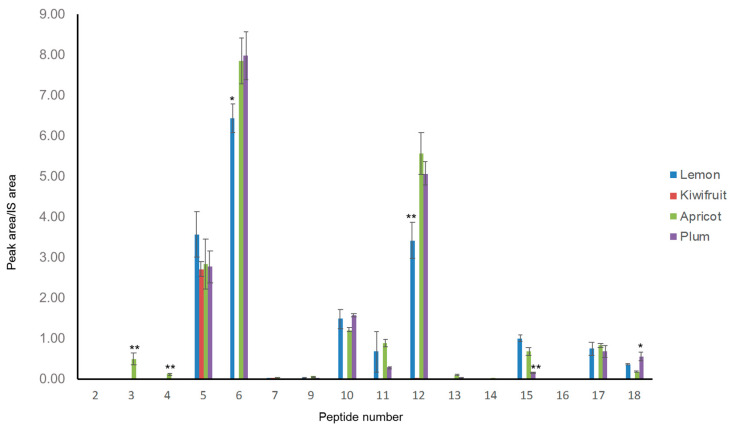
Area measured for peaks of the marker peptides in the LC-MS/MS analysis of four different Spirulina-enriched sorbets (lemon, kiwifruit, apricot and plum). Measurements were performed in triplicate on two different samples (*n* = 6): the graphs report the average areas and standard deviations. * *p* ≤ 0.05 and ** *p* ≤ 0.01 indicate a significant difference between that peptide abundance and the same peptide in the other three samples.

Three out of the four analyzed sorbets showed quite similar patterns (Figure 5); indeed, the relative abundances observed for the different markers in the case of apricot, plum and lemon sorbets demonstrate that the addition of Spirulina powder to the food matrix plays a similar role in its digestion process. In particular, the digestion of these three Spirulina-enriched sorbets produced high levels of phycocyanin C peptides (markers 5, 6, 10 and 12). In the case of kiwifruit, however, very few peptides were detected and their areas were generally lower than those of peptides of other sorbets. Kiwifruit, one of the most commercialized fruits on the international market, is well-known for its high content of proteinase inhibitors [39], which could interfere with the activity of pepsin and pancreatin in the digestion process, thus causing a significant reduction in peptide production. Overall, these results demonstrated that, in the sorbet food matrix, the choice of the fruit plays a very critical role in the production of potentially bioactive peptides.

## 4. Conclusions

Functional foods are obtained by adding mixtures of bioactive compounds (or microorganisms) to everyday foods, thus combining beneficial effects, ease of intake and good organoleptic characteristics. However, functional foods do not always retain the health characteristics of biologically active molecules; in many cases, this depends on bioavailability problems. Unlike other classes of molecules, the oral bioavailability of peptides from functional foods mainly depends on their production in the gastrointestinal digestion process. Multiple factors play a critical role in such production. Indeed, the chemical characteristics of each food matrix may significantly influence the qualitative and quantitative composition of the resulting digestion mixtures. For example, protein-rich foods (i.e., yoghurt, ice cream or milk) often reduce the production of bioactive peptides, since the matrix proteins compete with the functional proteins for proteolytic enzymes. Moreover, the interaction of functional proteins with the compounds present in the food can prevent their proteolysis. However, there are few studies on the effect, in terms of bioactive peptide production, of the inclusion of proteins with beneficial health activities in different foods.

Here we evaluated whether Spirulina-enriched foods can be considered a good source of the potentially bioactive peptides typical of these microalgae. In particular, pasta and four different sorbets were investigated, using a combination of simulated digestion protocol and mass spectrometry quantitative analysis of the resulting mixtures. The results obtained suggest that the incorporation into food matrixes produces specific effects on the generation of different peptides, particularly modifying the levels of those belonging to the β-subunit of C phycocyanin. However, in all but one of the functional foods analyzed, these changes possibly have negligible effect on the overall beneficial effects of Spirulina, as high levels of peptides are still produced. Only the functionalized kiwifruit sorbet appears to be an unsatisfactory source of peptides from *Arthrospira platensis*, probably due to the particular chemical composition of the kiwifruit; in fact, this fruit contains high levels of protease inhibitors that probably interfere with the proteolysis of the microalgae proteins.

## Figures and Tables

**Table 1:** Peptides from different proteins of *Arthrospira platensis* selected for the quantitative mass spectrometry-based method. Monitored *m*/*z* of parent and fragment ions are reported.

*N*	Protein	Peptide	Amino Acid Sequence	Precursor Ion	Product Ion
1	Allophycocyanin alpha chain	142–146	SGEDA	478.2	145.3
2	Allophycocyanin beta chain	10–16	NSSDVQG	706.3	503.4
3	Allophycocyanin beta chain	12–16	SDVQG	505.2	204.3
4	C-phycocyanin alpha subunit	10–16	SIADSQG	677.3	291.3
5	C-phycocyanin beta subunit	22–26	AQIDA	517.3	229.3
6	C-phycocyanin beta subunit	119–126	ALGTPGSS ^1^	689.3	347.3
7	Allophycocyanin alpha chain	62–72	RPDVVSPGGNA	534.8 *	654.4
8	Allophycocyanin beta chain	62–69	YSDITRPG	454.7 *	430.7
9	Allophycocyanin beta chain	1–6	-MQDAIT ^1^	678.3	300.6
10	C-phycocyanin beta subunit	127–132	VAVGVG	501.3	327.4
11	C-phycocyanin alpha subunit	100–107	IAGGTGPM	703.3	439.5
12	C-phycocyanin beta subunit	118–126	LALGTPGSS ^1^	802.4	347.5
13	C-phycocyanin alpha subunit	122–127	FELSPS ^1^	679.3	277.5
14	C-phycocyanin alpha subunit	99–107	LIAGGTGPM	816.4	590.5
15	Allophycocyanin alpha chain	118–123	SLGTPI ^1^	587.3	229.4
16	C-phycocyanin alpha subunit	121–127	TFELSPS ^1^	780.4	57.4
17	Allophycocyanin beta chain	117–123	NSLGVPI ^1^	699.4	372.5
18	Allophycocyanin alpha chain	95–104	GIVAGDVTPI ^1^	941.5	713.6

^1^ Potentially bioactive peptide [14,34], (*: doubly charged ion).

## Data Availability

The data presented in this study are available in supplementary material here.

## Supplementary Materials

The following are available online at https://www.mdpi.com/article/10.3390/nu13113919/s1, Table S1: List of peptides derived from the simulated gastrointestinal digestion of Spirulina identified by nanoLC-hrMS/MS analysis and bioinformatics elaboration of the data. Different peptides were grouped based on the protein they belong to. Figure S1: The extracted proteins were analyzed on 12% SDS-PAGE Tris–glycine buffer, under reducing conditions, followed by Coomassie Brilliant Blue staining (a) and UV spectra (b) of the protein component extracted from three different Spirulina samples (A, B and C).
