# Supplementary material for: Food Matrices Affect the Peptides Produced during the Digestion of Arthrospira platensis-Based Functional Aliments
Source: Nutrients. 2021 Nov 1;13(11):3919. doi: 10.3390/nu13113919 (PMC8625638; doi:10.3390/nu13113919)
Supplement: Supplementary file 1 [file nutrients-13-03919-s001.zip › nutrients-1414519-supplementary.pdf]

Supplementary information

Food matrices affect the peptides production during the digestion of Arthrospira platensis-based functional aliments

Giuliana Donadio, Valentina Santoro, Fabrizio Dal Piaz, Nunziatina De Tommasi*

Table S1: List of peptides derived from the simulated gastrointestinal digestion of spirulina identified by nanoLC-hrMS/MS analysis and bioinformatics elaboration of the data. Different peptides were grouped based on the protein they belong to.

Protein Gene: PHAA_ARTPT

Start	End	Observed	Mr(expt)	Mr(calc)	ppm	M	Score	Expect	Rank	U	Peptide
18	24	730.3611	729.3539	729.3545	-0.83	0	24	0.78	6	U	Y.LSPGELD.R
19	24	617.2776	616.2704	616.2704	-0.07	0	20	0.84	1	U	L.SPGELD.R
30	34	492.2309	491.2236	491.2227	1.78	0	31	0.36	1		F.VTSGE.R
62	72	534.7736	1067.5327	1067.536	-3.08	0	28	0.18	1	U	K.RPDVVSPGGNA.Y
95	99	416.2502	415.2429	415.2431	-0.36	0	16	0.57	1		Y.GIVAG.D
95	104	471.2687	940.5229	940.523	-0.11	0	66	0.0041	1	U	Y.GIVAGDVTPI.E
105	109	546.2757	545.2684	545.2697	-2.32	0	14	0.8	1		I.EEIGV.V
107	111	444.2814	443.2742	443.2744	-0.55	0	23	0.94	1		E.IGVVG.V
108	112	430.2659	429.2587	429.2588	-0.24	0	22	0.68	1		I.GVVGV.R
117	123	358.2208	714.427	714.4276	-0.76	0	25	1.8	6	U	Y.KSLGTPI.E
118	123	587.3398	586.3325	586.3326	-0.2	0	18	0.84	1	U	K.SLGTPI.E
118	124	716.3804	715.3732	715.3752	-2.88	0	16	1.2	3	U	K.SLGTPIE.A
142	146	478.1786	477.1713	477.1707	1.35	0	33	0.083	1		L.SGEDA.A

Protein gene: APCB_ARTPT

Start	End	Observed	Mr(expt)	Mr(calc)	ppm	M	Score	Expect	Rank	U	Peptide
1	6	678.3116	677.3043	677.3054	-1.64	0	27	1.3	3	U	-.MQDAIT.S
10	16	706.3016	705.2943	705.293	1.91	0	18	1.8	10	U	I.NSSDVQG.K
12	16	505.2265	504.2192	504.218	2.37	0	19	0.66	3	U	S.SDVQG.K
62	69	454.7265	907.4384	907.4399	-1.71	0	52	0.0043	1	U	L.YSDITRPG.G
63	69	373.1959	744.3772	744.3766	0.83	0	32	7	9	U	Y.SDITRPG.G
98	102	446.1881	445.1809	445.1809	-0.029	0	20	0.51	1		L.AGDPS.I
117	123	699.4024	698.3951	698.3963	-1.66	0	24	0.98	1	U	Y.NSLGVPI.G
135	139	476.2353	475.228	475.2278	0.39	0	12	0.56	1		K.EVTAG.L

139	143	416.2497	415.2424	415.2431	-1.61	0	26	0.14	1		A.GLVGA.D

Protein gene: Phycoalfa_ARTPT

Start	End	Observed	Mr(expt)	Mr(calc)	ppm	M	Score	Expect	Rank	U	Peptide
10	14	492.2306	491.2233	491.2227	1.18	0	16	1.6	5		V.SIADS.Q
10	16	677.3084	676.3011	676.3028	-2.52	0	22	0.6	1	U	V.SIADSQG.R
19	23	536.2566	535.2493	535.2489	0.69	0	7	1.1	2		F.LSSTE.I
52	56	418.2304	417.2231	417.2223	1.91	0	22	0.59	1		L.ISGAA.Q
66	73	446.6953	891.3761	891.3756	0.55	0	18	3.1	6	U	Y.TTQMQGPN.Y + Oxidation (M)
99	107	816.4274	815.4202	815.4211	-1.19	0	22	0.57	1	U	C.LIAGGTGPM.D
100	107	703.3455	702.3382	702.3371	1.68	0	21	2.9	4	U	L.IAGGTGPM.D
101	107	590.2595	589.2522	589.253	-1.39	0	37	1.2	4	U	I.AGGTGPM.D
102	107	519.2229	518.2156	518.2159	-0.53	0	17	1.3	5	U	A.GGTGPM.D
111	115	486.3282	485.3209	485.3213	-0.78	0	35	0.067	1		Y.LIAGI.D
112	117	617.3143	616.307	616.3068	0.33	0	45	0.28	1		L.IAGIDE.I
121	127	780.3781	779.3708	779.3701	0.9	0	28	2.8	4	U	R.TFELSPS.W
122	127	679.3305	678.3232	678.3224	1.15	0	29	0.82	1	U	T.FELSPS.W
123	127	532.2578	531.2505	531.254	-6.69	0	12	0.21	1		F.ELSPS.W
143	147	420.1724	419.1651	419.1652	-0.18	0	19	0.58	1		L.SGDAA.T

Protein gene: Phycobeta_ARTPT

5	10	340.7022	679.3898	679.3905	-1	0	24	0.75	1	U	A.FTKVVS.Q
5	12	440.2499	878.4853	878.4862	-0.97	0	33	0.94	1	U	A.FTKVVSQA.D
6	12	366.7152	731.4159	731.4178	-2.5	0	25	1.6	4	U	F.TKVVSQA.D
12	18	390.1732	778.3319	778.3279	5.14	0	30	0.53	1	U	Q.ADTRGEM.L
13	18	354.6524	707.2903	707.2908	-0.81	0	25	1.4	4	U	A.DTRGEM.L
13	19	419.1916	836.3687	836.3698	-1.34	0	28	1.2	3	U	A.DTRGEML.S + Oxidation (M)
19	23	519.2764	518.2692	518.27	-1.67	0	19	1	1	U	M.LSTAQ.I

22	26	517.2618	516.2545	516.2544	0.27	0	24	0.57	1		T.AQIDA.L
22	27	630.345	629.3377	629.3384	-1.16	0	34	0.58	1	U	T.AQIDAL.S
31	38	458.7633	915.5121	915.5137	-1.78	0	31	1.6	5	U	M.VAESNKRL.D
31	42	658.3506	1314.6867	1314.6891	-1.83	0	35	0.047	1	U	M.VAESNKRLDAVN.R
41	48	437.7398	873.4651	873.4668	-2	0	29	0.73	1	U	A.VNRITSNA.S
43	50	425.2246	848.4346	848.4352	-0.68	0	31	1.4	3	U	N.RITSNAST.I
49	55	691.3605	690.3532	690.3548	-2.34	0	23	0.53	1	U	A.STIVSNA.A
51	55	503.2811	502.2738	502.2751	-2.61	0	18	1.3	4		T.IVSNA.A
61	66	685.3498	684.3425	684.3442	-2.52	0	26	1.3	7	U	F.AEQPQL.I
91	95	351.1842	700.3538	700.3544	-0.89	0	15	0.77	1		L.RYVTY.A
113	117	341.1817	680.3489	680.3493	-0.6	0	19	0.77	1		G.LRETY.L
118	126	802.431	801.4238	801.4232	0.65	0	27	0.42	1	U	Y.LALGTPGSS.V
119	126	689.3442	688.337	688.3392	-3.21	0	29	0.89	4	U	L.ALGTPGSS.V
127	132	501.303	500.2957	500.2959	-0.33	0	34	0.37	1	U	S.VAVGVG.K
127	136	339.8632	1016.5677	1016.5688	-1.09	0	19	1.2	2	U	S.VAVGVGKMKE.A
129	136	424.2385	846.4624	846.4633	-1.04	0	43	0.097	1	U	A.VGVGKMKE.A
140	144	531.2756	530.2683	530.27	-3.25	0	21	0.52	1	U	L.AIVND.P

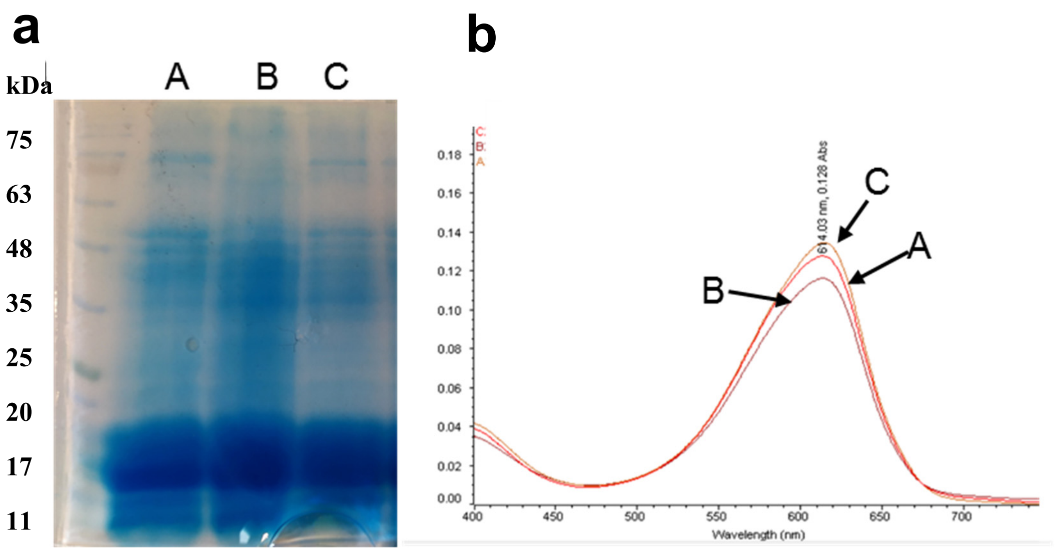

Figure S1. The extracted proteins were analyzed on 12% SDS-PAGE Tris–glycine buffer, under reducing conditions, followed by Coomassie Brilliant Blue staining (a) and UV spectra (b) of the protein component extracted from three different Spirulina samples (A, B and C).
